# Supplementary material for: Tumor-mediated microbiota alteration impairs synaptic tagging/capture in the hippocampal CA1 area via IL-1β production
Source: Commun Biol. 2023 Jul 3;6:685. doi: 10.1038/s42003-023-05036-1 (PMC10318068; doi:10.1038/s42003-023-05036-1)
Supplement: Supplementary file 2 — Supplementary Information [file 42003_2023_5036_MOESM2_ESM.pdf]

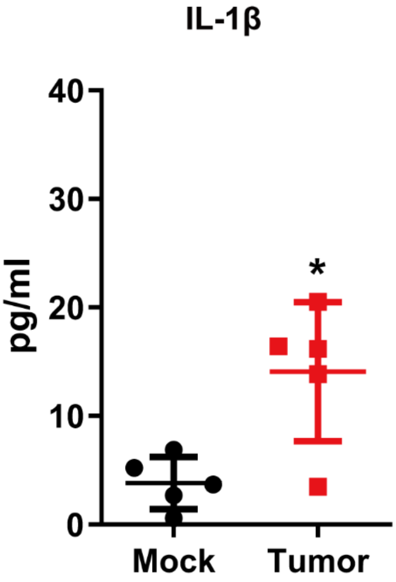

Supplementary Figure 1: HCC tumor-bearing mice had higher IL-1β in the serum. Mice were injected with either PBS or Hepa1-6 into the liver. Two weeks later, mice were scarified, serum were collected, the IL-1β level in the serum was quantified by ELISA (n=5). Error bars indicate ± SEM. *$p<0.05$.

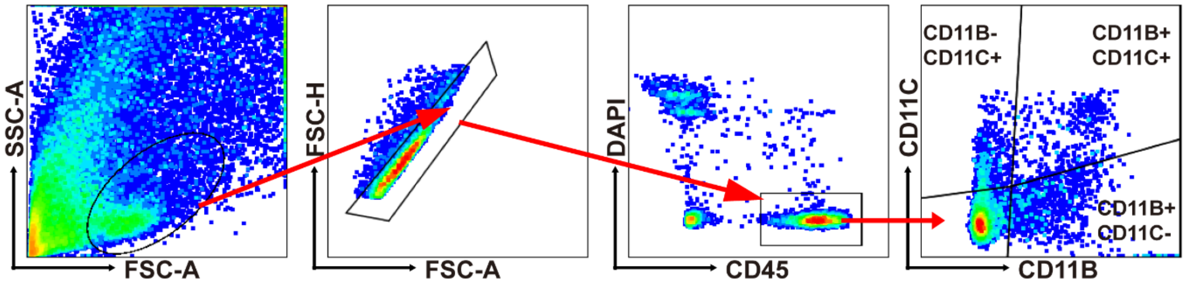

Supplementary Figure 2: Flow gating strategy for LP cells. Intestinal LP cells were isolated and stained with CD45, DAPI, CD11B and CD11C antibodies. The gating strategy was shown.
