# Supplementary material for: Tumor-mediated microbiota alteration impairs synaptic tagging/capture in the hippocampal CA1 area via IL-1β production
Source: Commun Biol. 2023 Jul 3;6:685. doi: 10.1038/s42003-023-05036-1 (PMC10318068; doi:10.1038/s42003-023-05036-1)
Supplement: Supplementary file 3 — Description of Additional Supplementary Files [file 42003_2023_5036_MOESM3_ESM.pdf]

Description of Additional Supplementary Files

File name: Supplementary Data

Description: The source data behind the graphs in the paper.
