# Supplementary material for: Tumor-mediated microbiota alteration impairs synaptic tagging/capture in the hippocampal CA1 area via IL-1β production
Source: Commun Biol. 2023 Jul 3;6:685. doi: 10.1038/s42003-023-05036-1 (PMC10318068; doi:10.1038/s42003-023-05036-1)
Supplement: Supplementary file 5 — Reporting Summary [file 42003_2023_5036_MOESM5_ESM.pdf]

# nature portfolio

Corresponding author(s): COMMSBIO-22-2314A

Last updated by author(s): May 26, 2023

# Reporting Summary

Nature Portfolio wishes to improve the reproducibility of the work that we publish. This form provides structure for consistency and transparency in reporting. For further information on Nature Portfolio policies, see our Editorial Policies and the Editorial Policy Checklist.

## Statistics

For all statistical analyses, confirm that the following items are present in the figure legend, table legend, main text, or Methods section.

| n/a | Confirmed | |
|---|---|---|
| ☐ | ☒ | The exact sample size ($n$) for each experimental group/condition, given as a discrete number and unit of measurement |
| ☐ | ☒ | A statement on whether measurements were taken from distinct samples or whether the same sample was measured repeatedly |
| ☐ | ☒ | The statistical test(s) used AND whether they are one- or two-sided<br>*Only common tests should be described solely by name; describe more complex techniques in the Methods section.* |
| ☒ | ☐ | A description of all covariates tested |
| ☒ | ☐ | A description of any assumptions or corrections, such as tests of normality and adjustment for multiple comparisons |
| ☐ | ☒ | A full description of the statistical parameters including central tendency (e.g. means) or other basic estimates (e.g. regression coefficient) AND variation (e.g. standard deviation) or associated estimates of uncertainty (e.g. confidence intervals) |
| ☒ | ☐ | For null hypothesis testing, the test statistic (e.g. $F$, $t$, $r$) with confidence intervals, effect sizes, degrees of freedom and $P$ value noted<br>*Give P values as exact values whenever suitable.* |
| ☒ | ☐ | For Bayesian analysis, information on the choice of priors and Markov chain Monte Carlo settings |
| ☒ | ☐ | For hierarchical and complex designs, identification of the appropriate level for tests and full reporting of outcomes |
| ☒ | ☐ | Estimates of effect sizes (e.g. Cohen's $d$, Pearson's $r$), indicating how they were calculated |

*Our web collection on statistics for biologists contains articles on many of the points above.*

## Software and code

Policy information about availability of computer code

| | |
|---|---|
| Data collection | NA |
| Data analysis | NA |

For manuscripts utilizing custom algorithms or software that are central to the research but not yet described in published literature, software must be made available to editors and reviewers. We strongly encourage code deposition in a community repository (e.g. GitHub). See the Nature Portfolio guidelines for submitting code & software for further information.

## Data

Policy information about availability of data

All manuscripts must include a data availability statement. This statement should provide the following information, where applicable:

- Accession codes, unique identifiers, or web links for publicly available datasets
- A description of any restrictions on data availability
- For clinical datasets or third party data, please ensure that the statement adheres to our policy

The 16s sequencing data that support the findings has been deposited to Genome Sequence Archive (GSA) under Accession ID: CRA009942.

## Human research participants

Policy information about studies involving human research participants and Sex and Gender in Research.

| | |
|---|---|
| Reporting on sex and gender | NA |
| Population characteristics | NA |
| Recruitment | NA |
| Ethics oversight | *Identify the organization(s) that approved the study protocol.* |

Note that full information on the approval of the study protocol must also be provided in the manuscript.

# Field-specific reporting

Please select the one below that is the best fit for your research. If you are not sure, read the appropriate sections before making your selection.

☒ Life sciences ☐ Behavioural & social sciences ☐ Ecological, evolutionary & environmental sciences

For a reference copy of the document with all sections, see nature.com/documents/nr-reporting-summary-flat.pdf

# Life sciences study design

All studies must disclose on these points even when the disclosure is negative.

| | |
|---|---|
| Sample size | For murine HCC models, each group contains 5-10 mice. |
| Data exclusions | No data was excluded from analyses. |
| Replication | All the experiments using cell lines and mice were repeat for three times. Numbers of replications in different experiments can be found in the corresponding figure legends. |
| Randomization | For ABX treatment or IL-1b depletion, mice were randomly divided into two groups. |
| Blinding | The cognitive function analysis was blinded as the researchers didn't know the mice belongs to which group. |

# Reporting for specific materials, systems and methods

We require information from authors about some types of materials, experimental systems and methods used in many studies. Here, indicate whether each material, system or method listed is relevant to your study. If you are not sure if a list item applies to your research, read the appropriate section before selecting a response.

## Materials & experimental systems

| n/a | Involved in the study | |
|---|---|---|
| ☐ | ☒ | Antibodies |
| ☐ | ☒ | Eukaryotic cell lines |
| ☒ | ☐ | Palaeontology and archaeology |
| ☐ | ☒ | Animals and other organisms |
| ☒ | ☐ | Clinical data |
| ☒ | ☐ | Dual use research of concern |

## Methods

| n/a | Involved in the study | |
|---|---|---|
| ☒ | ☐ | ChIP-seq |
| ☐ | ☒ | Flow cytometry |
| ☒ | ☐ | MRI-based neuroimaging |

## Antibodies

| | |
|---|---|
| Antibodies used | BUV395 Rat Anti-mouse CD45, Clone: 30-F11, Cat. 564279, BD Bioscience. PerCP/Cy5.5 Armenian Hamster Anti-mouse CD11C, Clone: N418, Cat. 117328, Biolegend. PE/Cy7 Rat anti-Human/Mouse CD11B, Clone: M1/70, Cat. 101216, Biolegend. |
| Validation | Antibodies were fully validated in biological samples according to the manufacturer's website. |

# Eukaryotic cell lines

Policy information about cell lines and Sex and Gender in Research

| | |
|---|---|
| Cell line source(s) | The primary cells were all isolated from male mice. |
| Authentication | The murine tumor cell line Hepa1-6 has been authenticated. |
| Mycoplasma contamination | All the cell line used in this study has been tested as mycoplasma negative by the Department of Comparative Medicine, National University of Singapore. |
| Commonly misidentified lines (See ICLAC register) | NA |

# Animals and other research organisms

Policy information about studies involving animals; ARRIVE guidelines recommended for reporting animal research, and Sex and Gender in Research

| | |
|---|---|
| Laboratory animals | C57BL/6 mice used in this study were all male. |
| Wild animals | NA |
| Reporting on sex | NA |
| Field-collected samples | NA |
| Ethics oversight | All animal studies were approved by the National University of Singapore Institutional Animal Care and Use Committee under protocol number R15-1041. |

Note that full information on the approval of the study protocol must also be provided in the manuscript.

# Flow Cytometry

## Plots

Confirm that:

☒ The axis labels state the marker and fluorochrome used (e.g. CD4-FITC).

☒ The axis scales are clearly visible. Include numbers along axes only for bottom left plot of group (a 'group' is an analysis of identical markers).

☒ All plots are contour plots with outliers or pseudocolor plots.

☒ A numerical value for number of cells or percentage (with statistics) is provided.

## Methodology

| | |
|---|---|
| Sample preparation | Mice were sacrificed and the small intestine was removed by cutting below the stomach and above the caecum. Intestinal contents were cleared by flushing with PBS. Intestines were cut into small pieces and put into 40 ml digestion buffer containing 5 Mm EDTA, 1mM DTT, 0.2 g dispase, and then shake for 40 min at 200 rpm, 37 degree. After incubation, filter the cell solution through a 40 µm cell strainer and pellet the cells by centrifugation. The cells were further applied with percoll gradient separation to isolate the leukocytes. Cells were stained with DAPI (Thermo Scientific), PerCP/Cy5.5-anti-mouse CD11c (clone: N418, Biolegend), PE-Cy7-anti-mouse CD11b (clone: M1/70, Biolegend) for 30 min before sorted on the BD FACSAria™ Fusion Flow Cytometers (BD Biosciences). |
| Instrument | BD FACSAria™ Fusion Flow Cytometers |
| Software | Flowjo_V10 was used to analyse the FACS data. |
| Cell population abundance | For FACS staining, at least 1 X 10^5 cells for each sample were acquired. For cell culture and cell sorting, the purity has been checked by FACS and only be used for experiments when the purity reached > 95%. |
| Gating strategy | The gating strategy has been shown in the supplementary Figure 2. |

☒ Tick this box to confirm that a figure exemplifying the gating strategy is provided in the Supplementary Information.
